# Prediction of poor exposure in endoscopic mitral valve surgery using computed tomography

**DOI:** 10.1093/ejcts/ezae070

**Published:** 2024-02-28

**Authors:** Yochun Jung, Sander M J van Kuijk, Hester Gietema, Jos G Maessen, Peyman Sardari Nia

**Affiliations:** Department of Thoracic and Cardiovascular Surgery, Chonnam National University Hospital, Chonnam National University School of Medicine, Gwangju, Republic of Korea; Department of Cardiothoracic Surgery, Maastricht University Medical Center, Maastricht, Netherlands; Department of Clinical Epidemiology and Medical Technology Assessment, Maastricht University Medical Center, Maastricht, Netherlands; Department of Radiology and Nuclear Medicine, Maastricht University Medical Center, Maastricht, Netherlands; Department of Cardiothoracic Surgery, Maastricht University Medical Center, Maastricht, Netherlands; Department of Cardiothoracic Surgery, Maastricht University Medical Center, Maastricht, Netherlands

**Keywords:** Endoscopic, Mitral valve, Exposure

## Abstract

**OBJECTIVES:**

In endoscopic mitral valve surgery, optimal exposure is crucial. This study aims to develop a predictive model for poor mitral valve exposure in endoscopic surgery, utilizing preoperative body profiles and computed tomography images.

**METHODS:**

We enrolled patients undergoing endoscopic mitral valve surgery with available operative video and preoperative computed tomography. The degree of valve exposure was graded into 0 (excellent), 1 (fair), 2 (poor) and 3 (very poor). Intrathoracic dimensions–anteroposterior width (chest anteroposterior) and left-to-right width (chest width) of the thorax, height of right hemi-thorax (chest height), angle between the left ventricular axis and the horizontal plane (left ventricle apex angle), heart width, level of diaphragm in midline, and vertical distance between the midline diaphragm level and the highest top of the right diaphragm (Δdiaphragm) were measured.

**RESULTS:**

Among 263 patients, mitral valve exposure was graded as 0 in 131 (49.8%), 1 in 72 (27.4%), 2 in 46 (17.5%) and 3 in 14 (5.3%). Body mass index, chest width, left ventricle apex angle, heart width and Δdiaphragm were identified as independent predictors of grades 2 and 3 exposure by stepwise logistic regression analysis, with an area under the receiver operating characteristic curve of 0.822 (*P* < 0.001). Univariate logistic regression for grade 3 exposure prediction revealed that Δdiaphragm had the largest area under the curve (0.826, *P* < 0.001).

**CONCLUSIONS:**

Poor mitral valve exposure occurred in approximately one-fourth of the endoscopic surgery series and might be predicted preoperatively using body mass index and computed tomography measurements to help determine the surgical approach.

**Figure:**
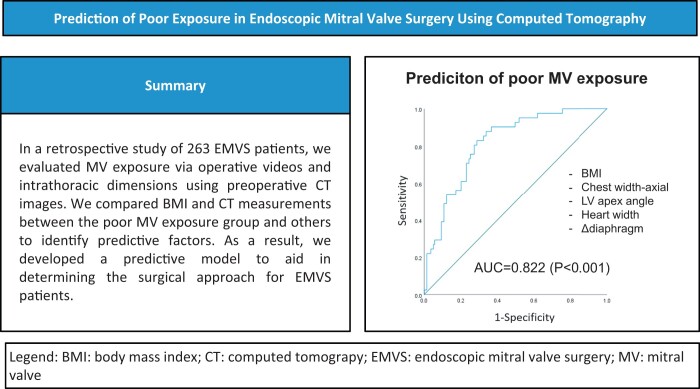

## INTRODUCTION

Compared to conventional mitral valve (MV) surgery through sternotomy, endoscopic mitral valve surgery (EMVS) through mini-thoracotomy has a faster recovery, shorter hospital stay and better cosmetic effects [1–3]. In EMVS, optimal exposure is crucial because compared to sternotomy, the size of the working port is much smaller and the space in which the surgical instruments can be moved is greatly limited [4]. Although EMVS can provide unobstructed indirect vision for MV, it does not guarantee a good surgical exposure in all cases. Often, a part of the left atrial wall around the mitral annulus collapses into the cavity and enters the field of view [5], and the annular shape is deformed by external compression. This limits exposure of the MV, which makes it difficult to evaluate the annular size or range of leaflet prolapse during valve analysis and makes it difficult to suture the annulus or leaflet. Such difficulties can lead to prolonged surgical time or unsatisfactory MV repair results, especially at the hands of inexperienced surgeons. Therefore, preoperative prediction of poor MV exposure would be useful to determine the surgical approach. However, few studies have investigated how often poor MV exposure occurs in patients undergoing EMVS and whether useful predictor exists.

If the approach to the MV is constant during EMVS, the difference in MV exposure will likely originate from the difference in anatomical structure in the thoracic cavity. The patient’s body profile (e.g. height and weight) is the most easily obtainable index that indirectly reflects the difference in the thoracic structure. Computed tomography (CT) can directly and noninvasively provide various dimensions of the heart and thoracic structure. Recent technological advances of CT hardware and software have improved its spatial and temporal resolution, making it a standard preoperative examination for cardiac surgery [6, 7]. Therefore, we aimed to develop a prediction model of poor MV exposure in EMVS using preoperative patients’ body profiles and the dimensions of the heart and thoracic cage measured from chest CT images.

## PATIENTS AND METHODS

### Ethical statement

This study was approved by the Institutional Review Board of Maastricht University Medical Center, which waived the requirement for patient consent due to the retrospective nature of the study (IRB number: 2023-3615; approval date: 26 January 2023).

### Patients

We enrolled patients undergoing EMVS at Maastricht University Medical Center from December 2013 to December 2022, with both surgical video and preoperative chest CT available, excluding those with a history of mitral valve replacement (MVR) or ring annuloplasty. Their medical records, surgical videos and preoperative chest CT were retrospectively analysed. This cohort includes the full learning curve of the programme.

### Surgical exposure of the mitral valve

Our method of MV exposure has been described previously [8]. In all patients, the most ideal location for the incision was determined based on three-dimensional reconstructions of CT images, as previously described in studies [9, 10]. This ideal position entails aligning the incision with the interatrial groove. All procedures were performed endoscopically. Briefly, a right-sided mini-thoracotomy of 3–4 cm was performed in the 4th or 5th intercostal space based on three-dimensional CT reconstruction images. An Alexis soft-tissue retractor (Applied Medical, Rancho Santa Margarita, CA, USA) was inserted to prevent extensive rib retraction and reduce postoperative pain. Visualization was accomplished by a 10-mm 30° three-dimensional endoscope (Karl Storz Endoscopy-America, Culver City, CA, USA) placed through a trocar in the same intercostal space as the mini-thoracotomy. The camera-port was always in the same intercostal space below port-access incision so that the camera would give unobstructed view of the MV.

Once on cardiopulmonary bypass, the pericardium was opened anterior to the phrenic nerve, starting from the level of the ascending aorta to the level of the diaphragm. Multiple pericardial sutures were placed to retract the pericardium to achieve optimal exposure. Before aortic occlusion, a stab incision was made parasternally to place the external part of the atrial retractor (HV retractor, USB Medical, Huntingdon Valley, PA, USA). In all cases, the same atrial retractor was used. Subsequently, the left atrium was opened and the atrial wall was retracted anteriorly by inserting the blade of the atrial retractor and connecting it to the shaft of the outside table-mounted holder. The blade size was determined preoperatively, based on left atrial volume. A sump sucker connected to an additional vent was placed in the left atrium for adequate drainage.

### Visual grading of mitral valve exposure

**Figure 1:**
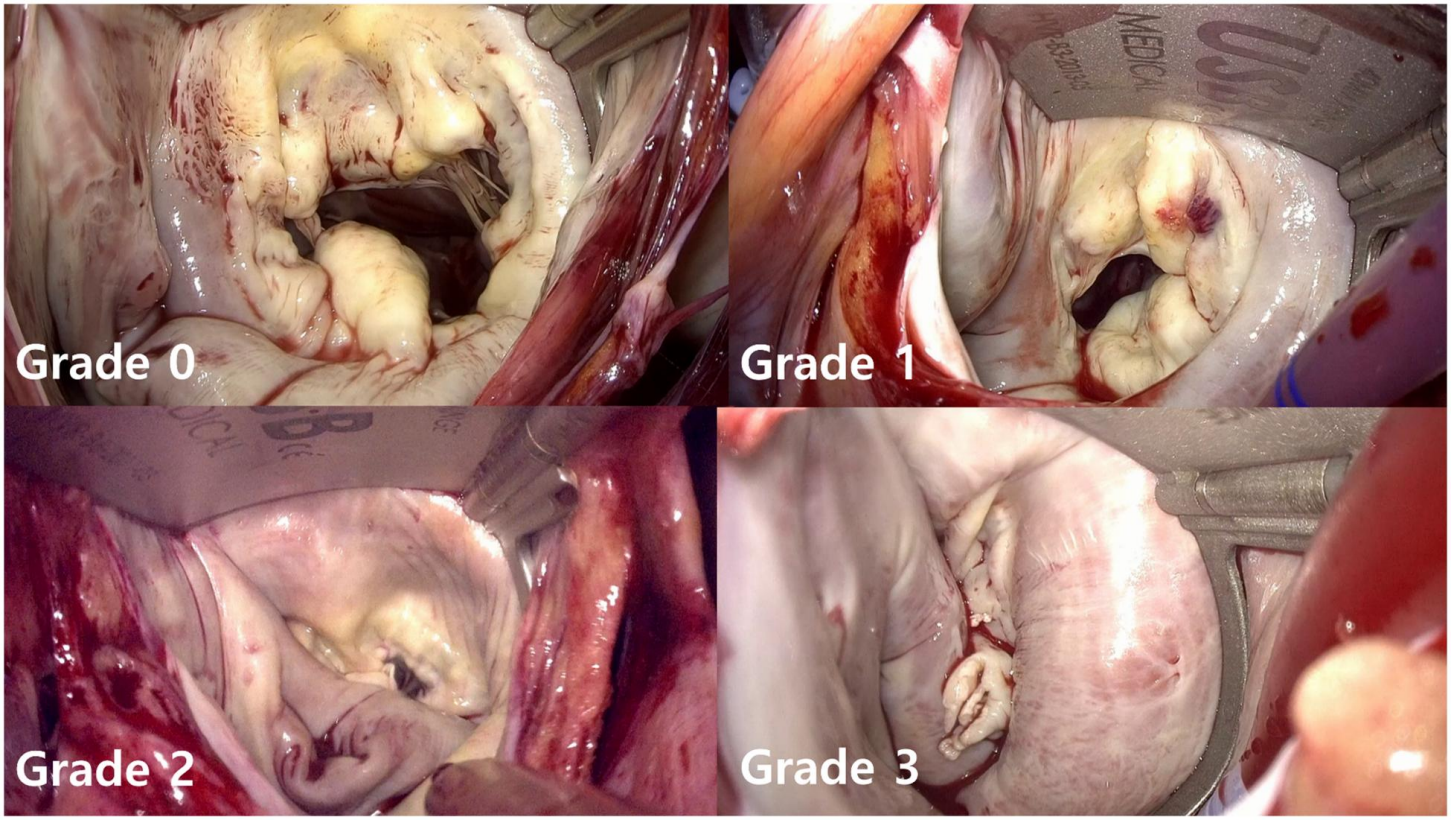
Grading of mitral valve exposure.

First, 2 MV surgeons reviewed 50 surgical videos to reach an agreement on the visual grading of MV exposure, and 1 reviewed the surgical videos and graded MV exposure of all study subjects. The grade of MV exposure was defined as follows (Fig. 1):

Grade 0 (excellent): all or most of the MV annulus is exposed and the annular shape is intact.
Grade 1 (fair): the MV annulus is partially covered by atrial wall or deformed due to compression or squeezing (i.e. the annulus is pressed up and down or left and right in the operator view, respectively), but it does not significantly interfere with the surgical procedure.
Grade 2 (poor): the MV annulus is obscured or deformed, which significantly interferes with the surgical procedure, although grade 3 exposure cases are excluded.
Grade 3 (very poor): the complete annular shape cannot be determined due to severe squeezing of the MV.

In this study, grade 2 or higher (grade 2 + 3) was defined as poor MV exposure.

### Measurements of intrathoracic dimensions

**Figure 2:**
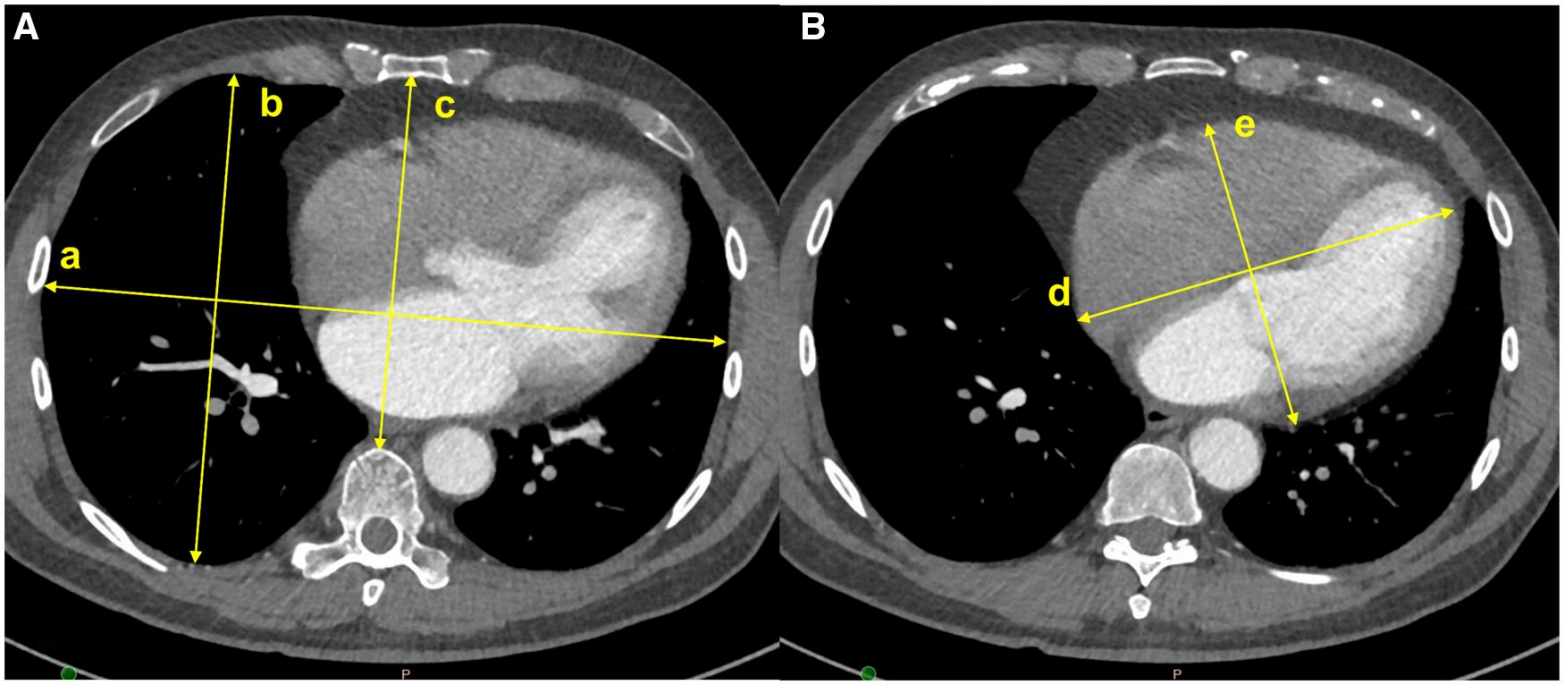
Measurements in axial images. (**A**) Image at the level in which the mitral valve looks widest. (a) Transversally widest dimension of the thorax (‘chest width–axial’). (b) Widest anteroposterior dimension in the right hemi-thorax (‘chest AP–axial’). (c) Anteroposterior dimension in midline (‘mid AP–axial’). (**B**) Image at the level in which the heart shadow looks widest. (d) Longest dimension of the heart (‘long heart–axial’). (e) Longest dimension of the heart vertical to the long heart–axial (‘short heart–axial’). AP: anteroposterior.

**Figure 3:**
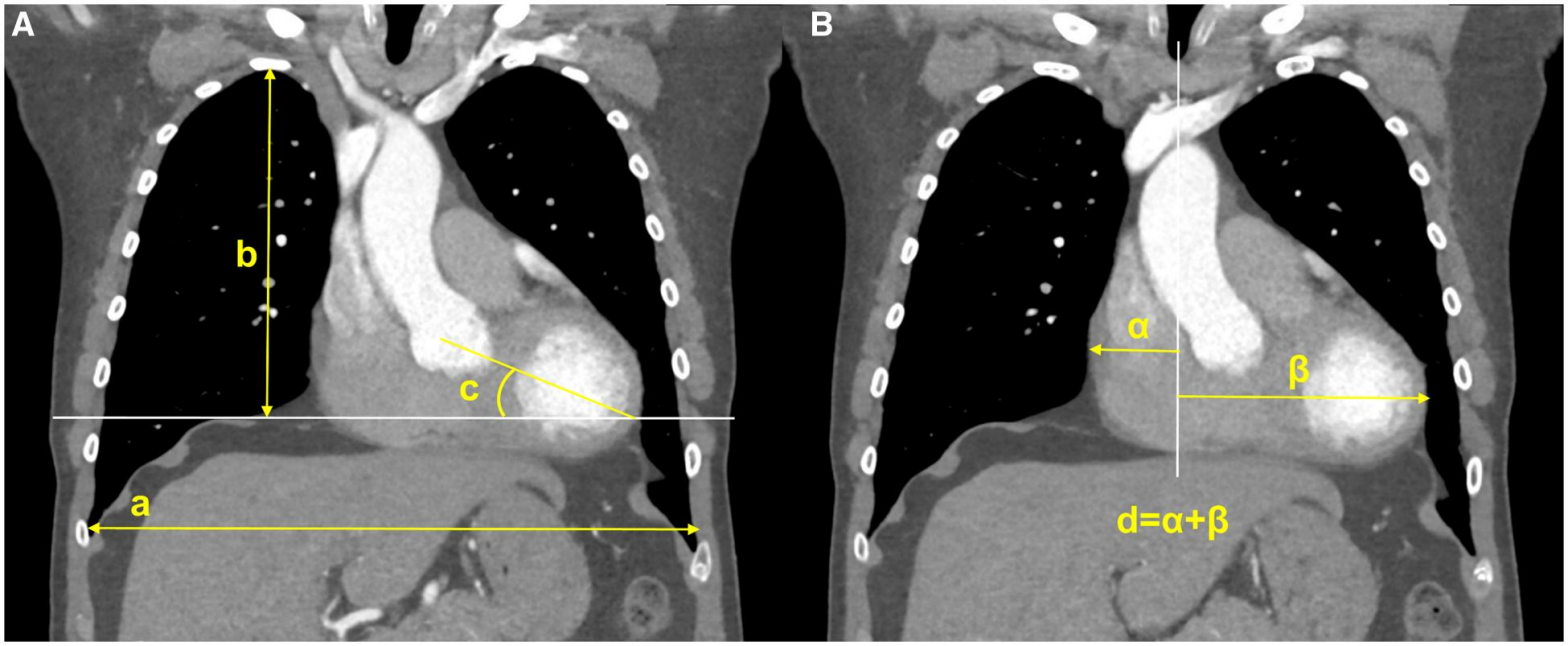
Measurements in coronal images. (**A**) Image at the level in which the aortic root and ascending aorta are transected most widely. (a) Transversally widest dimension of the thorax (‘chest width–coronal’). (b) Dimension between the top of the right diaphragm and the highest level of the thoracic inlet (‘chest height–coronal’). (c) Angle between the left ventricular axis and horizontal plane (‘LV apex angle’). (**B**) Image at the level in which the heart shadow looks widest. (d) Sum of the longest lengths from the vertical line in the middle of the mediastinum to the left and right heart borders (‘heart width’). LV: left ventricle.

**Figure 4:**
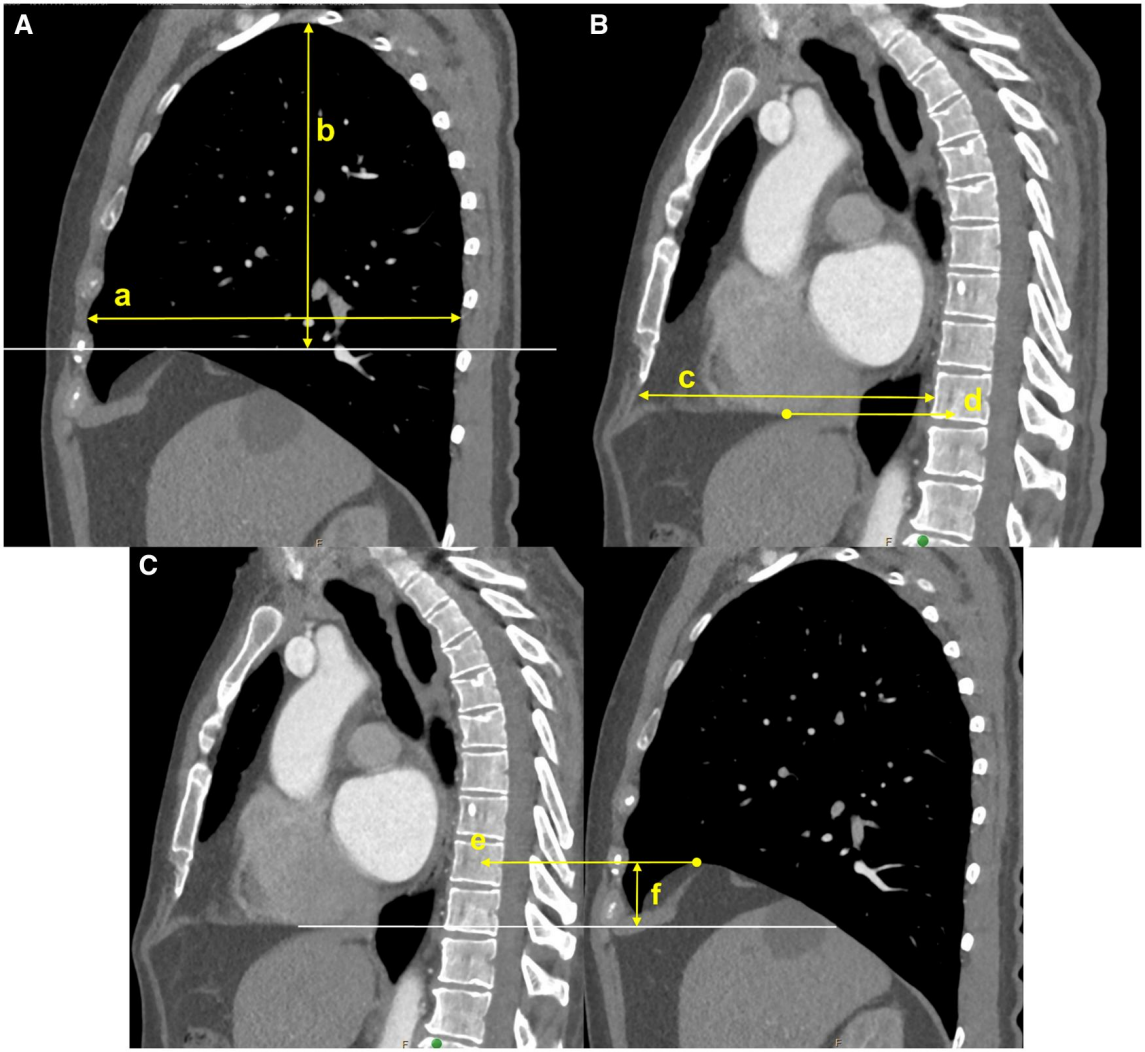
Measurements in sagittal images. (**A**) Image at the level in which the rib cage of the right hemi-thorax looks widest. (a) Widest anteroposterior dimension of the thorax (‘chest AP–sagittal’). (b) Dimension between the top of the right diaphragm and the highest level of the thoracic inlet (‘chest height–sagittal’). (**B**) Image at midline (in which the spinal canal is widest). (c) Widest anteroposterior dimension of the thorax (‘mid AP–sagittal’). (d) Level of the diaphragm according to the number of the thoracic spine (‘mid diaphragm’; in this example, the mid diaphragm is 11.5 designating a level between T11 and T12). (**C**) Images at the midline and the level at which the top of the right diaphragm is highest. (e) Level of the top of the right diaphragm according to the number of the thoracic spine (‘right diaphragm’; in this example, the right diaphragm is 10 designating a level of T10). (f) Vertical distance between the midline diaphragm level and the highest top of the right diaphragm (‘Δdiaphragm’). AP: anteroposterior.

Two cardiac surgeons determined the intrathoracic dimensions that might affect MV exposure, before a radiologist defined them further. Consequently, 5, 4 and 6 dimensions were defined in the axial, coronal and sagittal view images, respectively (Figs 2–4). Despite differences in the examination protocol depending on the hospital where the CT was performed, all cases with CT images covering the thoracic cage, performed within 1 year before surgery, were enrolled, regardless of the examination protocol. Consequently, some dimensions could not be measured because specific view images were unavailable, and these missing values were excluded from statistical analysis. All images were reviewed using a NilRead Diagnostic Image Viewer (Hyland Software Inc., Westlake, OH, USA) and all dimensions were measured by a single surgeon using the measurement tools in the software.

### Effect of mitral valve exposure on mitral valve repair

Among patients who underwent MV repair, we compared the differences in adverse outcomes [sternotomy conversion due to poor MV exposure, operative mortality, postoperative mitral regurgitation (MR), postoperative mitral stenosis (MS) and MV reoperation] between the patient group with grade 2 + 3 or grade 3 exposure and the other groups. There was no predefined follow-up protocol after discharge, and in cases where a stable condition was observed postoperatively, follow-up was conducted at the local hospital. When follow-up was performed at our hospital, the decision to conduct echocardiography was at the discretion of the attending physician. Based on the echocardiographic findings last performed at our hospital (immediately before reoperation in the case of MV reoperation), MR grade >1+ was defined as postoperative MR, and a transvalvular mean pressure gradient >5 mmHg was defined as postoperative MS [11].

### Statistical analysis

Preoperative body profile, CT measurements and clinical outcomes were compared between the grade 2 + 3 or grade 3 exposure group and the other groups. For continuous variables, histograms and Q–Q plots were generated to determine the normality of the distribution. Normally distributed data were summarized as means and standard deviations and compared using independent-samples Student’s *t*-test. Non-normal data were summarized as medians and ranges and compared using the Mann–Whitney *U*-test. Categorical variables were expressed as frequencies and percentages of cases and compared using either the χ^2^ test or Fisher’s exact test, as necessary. We chose not to impute missing CT values, as these were due to differences in local protocols of CT examinations, which were not likely associated with any patient’s characteristics.

Eighteen variables (3 of the patient’s body profile and 15 of intrathoracic dimensions measured in CT images) were used as candidate predictors to create a prediction model for poor MV exposure. First, we checked for multicollinearity by computing correlations for each pair of candidate predictors and by computing variance inflation factors for the complete set of predictors; those with a Pearson’s correlation coefficient (Spearman’s rank correlation coefficient in the case of variables with skewed distributions) >0.7 were identified (https://www.andrews.edu/∼calkins/math/edrm611/edrm05.htm). In cases where 2 variables showed a high correlation, the variable with fewer missing values and higher reproducibility of measurement was selected. We then created multiple sets of candidate predictors that only contained variables that were not highly correlated with each other. For each variable set, stepwise regression analysis was performed to predict poor MV exposure. We computed the area under the receiver operating characteristic curve, as a measure of discrimination. Area under the curve (AUC) values between models based on different variable sets were compared using the method of DeLong. Subsequently, the variable set with the largest AUC was selected as the final model, and for each variable remaining in the multivariable model (two-tailed nominal *P* < 0.20), the *P*-value, odds ratio and 95% confidence interval (CI) were calculated. To assess model calibration, we visually inspected the calibration plot, and computed the Hosmer and Lemeshow goodness-of-fit test statistic. The final prediction model was internally validated by standard bootstrapping techniques with 1000 bootstraps. To determine predictors of grade 3 exposure, we could only use univariable logistic regression analysis because, using our definition, the number of events was too low for multivariable modelling. Therefore, we performed univariable analysis with each variable of the previously selected variable set, and the optimal predictor was obtained by comparing the AUCs. The Kaplan–Meier method was used to assess the conditional probabilities of freedom from postoperative MR, MS and MV reoperation, and the log-rank test was used to determine inter-group differences. The analysis was conducted using SPSS Statistics for Windows version 28.0 (IBM Corp., Armonk, NY, USA) and R, a language for statistical computing, version 4.0.1.

## RESULTS

### Baseline characteristics

Among 387 patients who underwent EMVS, both surgical video and preoperative chest CT were available in 285 patients. After excluding 22 patients with a history of MVR or ring annuloplasty, 263 patients were finally included in this study. The mean age was 64.5 (with standard deviation of 10.6) years, and 165 patients (62.7%) were men. Preoperative diagnosis was MR, MS and MR + MS in 241 (91.6%), 16 (6.1%) and 5 patients (1.9%), respectively. One patient was diagnosed with MV vegetation only, without MV dysfunction. Fibroelastic deficiency was the most common cause of MR in 159 cases (66.0%), followed by Barlow’s disease in 43 cases (17.8%), functional in 22 cases (9.1%; atrial 16, ventricular 6) and infective endocarditis in 17 cases (7.1%). Twenty-two patients (8.4%) had a history of pericardiotomy for cardiac operation, among whom, 3 had undergone mini-thoracotomy (1 MV repair, 2 thoracoscopic ablation) and 4 had undergone MV procedure (1 MV repair, 2 open mitral commissurotomy and 1 beating heart transapical neochord procedure). Regarding the surgical procedures, MV repair was carried out in 231 cases, MVR in 31 cases and vegetation removal alone in 1 case. Among the 241 patients diagnosed with MR (including patients with reoperation and endocarditis) preoperatively, MV repair was performed in 231 patients, account for a repair rate of 95.9%.

### Mitral valve exposure grade

A total of 131 (49.8%), 72 (27.4%), 46 (17.5%) and 14 cases (5.3%) were classified into grades 0, 1, 2 and 3, respectively.

### Computed tomography measurements

Among all subjects, axial, coronal and sagittal view images were available in 254 (96.6%), 195 (74.1%) and 199 patients (75.7%), respectively. Some dimensions could not be measured due to the limitation of the CT scan range. The patients of the grade 2 + 3 exposure group had a larger body size, larger anteroposterior and left-to-right dimensions of the thoracic cage, lower height of the thoracic cage and higher diaphragm level compared to those of the other groups (Table 1). The short axis dimension of the heart shadow in axial view (short heart–axial) was larger in the grade 2 + 3 exposure group, but there was no significant difference in other variables of heart size (long heart–axial, heart width). Additionally, the grade 2 + 3 exposure group had a significantly smaller angle between the left ventricular axis and horizontal plane [left ventricle (LV) apex angle], as well as a longer vertical distance between the midline diaphragm level and the highest top of the right diaphragm (Δdiaphragm) (Table 1).

**Table 1:** Comparison of the poor mitral valve exposure group with others

Total (*n* = 263)	Grade 2 + 3 (*n* = 60)	Others (*n* = 203)	*P*-value
Age (year)	60, 64.9 (7.7)	203, 64.4 (11.4)	0.703
Male, *n*	60, 41 (68.3)	203, 124 (61.1)	0.308
Height (cm)	60, 173.5 (8.0)	203, 173.6 (10.3)	0.964
Weight (kg)	60, 85.5 (13.4)	203, 74.6 (13.5)	<0.001
BMI (kg/m^2^)	60, 28.5 (4.5)	203, 24.7 (3.7)	<0.001
Chest width–axial (mm)	56, 271.2 (23.2)	195, 264.1 (23.3)	0.045
Chest AP–axial (mm)	58, 199.2 (18.3)	196, 189.4 (22.6)	<0.001
Mid AP–axial (mm)	58, 140.4 (18.6)	196, 129.6 (21.6)	<0.001
Long heart–axial (mm)	58, 147.2 (17.6)	196, 143.0 (17.5)	0.111
Short heart–axial (mm)	58, 125.0 (11.6)	196, 117.7 (16.1)	<0.001
Chest width–coronal (mm)	44, 276.9 (32.6)	143, 267.8 (28.6)	0.076
Chest height–coronal (mm)	41, 152.1 (29.3)	133, 169.5 (28.7)	<0.001
LV apex angle (°)	45, 26.7 (8.0)	150, 33.4 (8.9)	<0.001
Heart width (mm)	44, 146.5 (17.7)	144, 145.3 (16.1)	0.688
Chest AP–sagittal (mm)	44, 204.4 (18.4)	146, 192.8 (22.3)	0.002
Chest height–sagittal (mm)	28, 184.2 (25.5)	103, 198.0 (26.6)	0.016
Mid AP–sagittal (mm)	44, 147.6 (16.6)	146, 135.9 (21.4)	<0.001
Mid diaphragm (T spine no)	45, 10.5 (8.0–12.0)	154, 11 (9.0–12.5)	<0.001
Right diaphragm (T spine no)	45, 9.5 (7.0–12)	154, 10.5 (7.5–12)	<0.001
Δdiaphragm (mm)	44, 25.5 (5–59)	149, 16 (-3–65)	0.003

Values are presented as number (%), mean (standard deviation) or median (range) with the number of cases in which values for the variable could be obtained.

AP: anteroposterior dimension; BMI: body mass index; LV: left ventricle; T spine no: thoracic spine number.

### Prediction of poor mitral valve exposure (grade 2 + 3)

**Figure 5:**
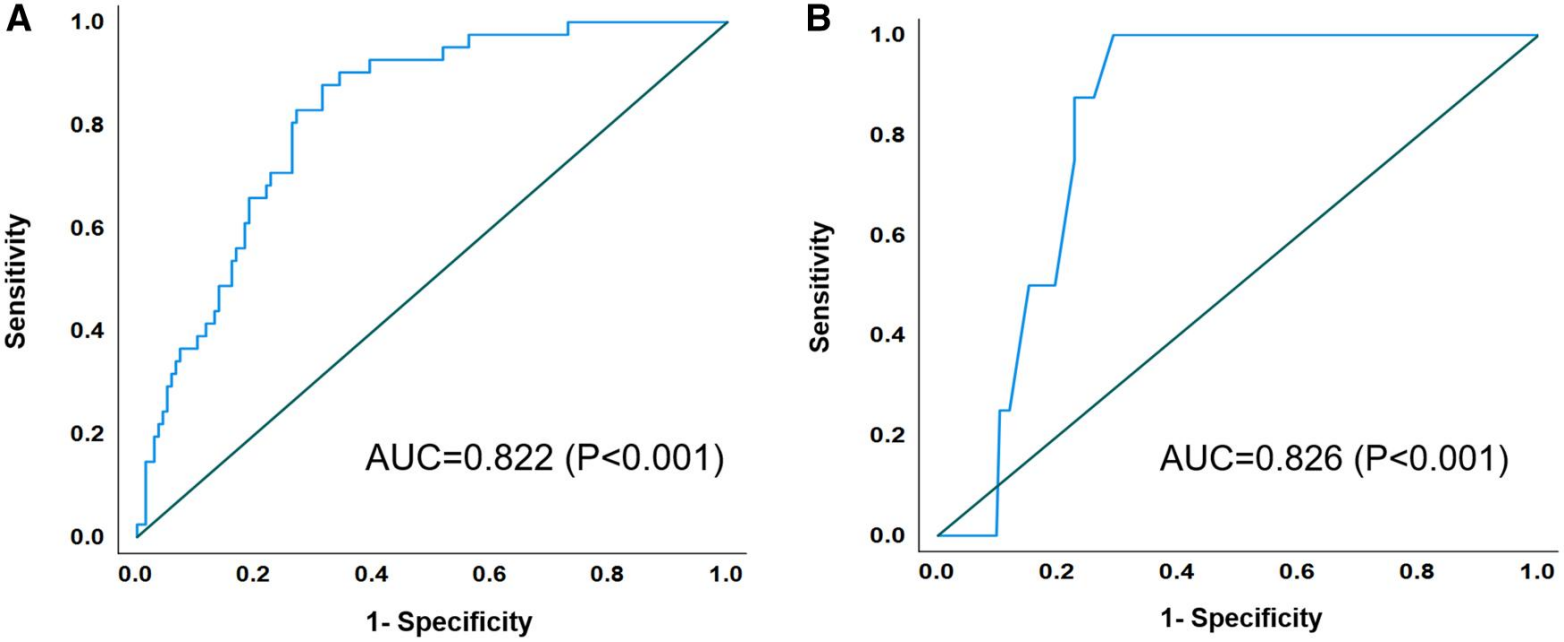
ROC curve of the poor mitral valve exposure prediction model. (**A**) Prediction of grade 2 + 3 exposure using 5 variables. (**B**) Prediction of grade 3 exposure using Δdiaphragm. AUC: area under the curve; ROC curve: receiver operating characteristic curve.

Correlation analysis of all variables identified the combinations of variables with a correlation coefficient >0.7; and among the variables showing high correlation, variables with fewer missing values and higher reproducibility of measurement were selected (Supplementary Material, Table S1). By combining the combinations of uncorrelated variables after correlation analysis with all other candidate predictors, 6 variable sets were obtained for multivariable logistic regression analysis (Supplementary Material, Table S2). None of the AUCs was significantly different from any of the others (range of *P*-values: 0.337–0.983). We selected a combination of body mass index (BMI), chest width–axial, chest anteroposterior–axial, chest height–coronal, LV apex angle, heart width, mid diaphragm and Δdiaphragm as the variable set with the largest observed AUC (Supplementary Material, Table S2). Stepwise regression analysis performed using this variable set revealed that BMI, chest width–axial, LV apex angle, heart width, and Δdiaphragm were the predictors of poor MV exposure (Table 2, Supplementary Material, Fig. S1), while receiver operating characteristic analysis showed an AUC of 0.822 (95% CI 0.757–0.886; *P* < 0.001) (Fig. 5A). The calibration plot shows good calibration in the range of probabilities that are observed most often, between 0.0 and 0.6, and some overestimation in predicted probabilities over 0.6 (Supplementary Material, Fig. S2). The Hosmer and Lemeshow test was not significant (*P* = 0.649), indicating no evidence of lack of model fit. Standard bootstrapping techniques with 1000 bootstraps to internally validate the model with 5 predictors revealed that the optimism in the AUC was 0.025, meaning that the optimism-corrected AUC (i.e. the expected AUC in future patients) would be 0.797.

**Table 2:** Predictors of poor mitral valve exposure (grade 2 + 3)

Variables	B	OR (95% confidence interval)	*P*-value
BMI	0.248	1.282 (1.132–1.450)	<0.001
Chest width–axial	0.013	1.013 (0.994–1.032)	0.188
LV apex angle	–0.072	0.931 (0.878–0.987)	0.017
Heart width	–0.027	0.973 (0.944–1.003)	0.077
Δdiaphragm	0.030	1.030 (0.996–1.065)	0.081
Intercept	–5.993	NA	NA

B: regression coefficient; BMI: body mass index; LV: left ventricle; NA: not applicable; OR: odds ratio.

### Prediction of grade 3 mitral valve exposure

Univariate logistic regression performed on each of the 8 variables of the previously selected set revealed that Δdiaphragm was the variable with the highest AUC [0.826 (95% CI 0.761–0.890); *P* < 0.001; Supplementary Material, Table S3, Fig. 5B].

### Effect of poor mitral valve exposure on the clinical outcome of mitral valve repair

Among the 241 patients diagnosed with preoperative MR, MVR was performed in 10 cases. Among these, MVR was performed without attempting MV repair in 8 patients: 4 had ischaemic MR, and 5 were deemed unsuitable for MV repair based on intraoperative valve examination (4 with poor anterior MV leaflet quality, 1 with infective endocarditis). The remaining 2 patients, both with infective endocarditis, underwent MVR after unsuccessful MV repair attempts. One of them underwent sternotomy conversion due to poor MV exposure (grade 2).

Among the 231 patients who underwent MV repair, sternotomy conversion due to poor surgical field of view occurred in 1 case in the grade 3 exposure group. Operative mortality was 4 cases (1.7%). The median follow-up duration of the remaining 227 patients was 190 days (5 days–9.1 years), during which, 14 patients (6.2%) underwent MV reoperation (4.0 per 100 person-years; postoperative MR: 13 and MS: 1). Postoperative echocardiography was performed in 226 patients (97.8%), 88 of whom only had a record of predischarge echocardiography. The median postoperative duration to the last echocardiography (before reoperation in the case of MV reoperation) was 103 days (1 day–9.0 years). Postoperative MR (grade >1+) was confirmed in 33 cases (14.3%; 9.0 per 100 person-years) and postoperative MS (mean pressure gradient >5 mmHg) in 28 cases (12.1%; 7.6 per 100 person-years).

The MR aetiology was similar between the grade 2 + 3 or grade 3 exposure group and the other groups. The overall adverse outcomes and postoperative MS were higher in the grade 2 + 3 exposure group, although not significantly. The one-year probabilities of freedom from postoperative MR, MS and MV reoperation showed no significant differences between the grade 2 + 3 exposure group and the other group [MR: 0.84 (95% CI 0.73–0.97) vs 0.87 (95% CI 0.81–0.94), *P* = 0.556; MS: 0.78 (95% CI 0.67–0.92) vs 0.90 (95% CI 0.85–0.95), *P* = 0.067; MV reoperation: 0.91 (95% CI 0.82–1.00) vs 0.93 (95% CI 0.88–0.99), *P* = 0.294]. However, the overall adverse outcomes, postoperative MS and MV reoperation were significantly higher in the grade 3 exposure group compared to the other groups (Table 3). The grade 3 exposure group exhibited a lower 1-year probability of freedom from postoperative MR than the other group, although not statistically significant [0.75 (95% CI 0.53–1.00) vs 0.88 (95% CI 0.82–0.94), *P* = 0.077]. Nevertheless, the one-year probabilities of freedom from postoperative MS and MV reoperation were significantly lower in the grade 3 exposure group compared to the other group [MS: 0.54 (95% CI 0.32–0.90) vs 0.89 (95% CI: 0.85–0.94), *P* < 0.001; MV reoperation: 0.82 (95% CI 0.62–1.00) vs 0.94 (95% CI 0.90–0.99), *P* = 0.030].

**Table 3:** Comparison of pathology and adverse outcomes between the grade 2 + 3/grade 3 exposure group and others

MV repair (*n* = 231)	Grade 2 + 3 (*n* = 59)	Others (*n* = 172)	*P*-value	Grade 3 (*n* = 14)	Others (*n* = 217)	*P*-value
Aetiology of MR						
Barlow	7 (11.9)	36 (20.9)	0.123	2 (14.3)	41 (18.9)	>0.999*
Functional	3 (5.1)	15 (8.7)	0.574*	0 (0)	18 (8.3)	0.609
Endocarditis	3 (5.1)	10 (5.8)	>0.999*	2 (14.3)	11 (5.1)	0.181*
Previous pericardiotomy	1 (1.7)	13 (7.6)	0.124*	0 (0)	14 (6.5)	>0.999
Postop interval until						
Last EchoCG	96 days (2 days–7.5 years)	103 days (1 day–9 years)	0.892	168 days (4 days–4 years)	96 days (1 day–9 years)	0.461 (0.108)
Last follow-up	317 days (7 days–7.5 years)	189 days (5 days–9.1 years)	0.781	1.3 years (7 days–5.5 years)	174 days (5 days–9.1 years)	
Adverse outcomes						
Overall	20 (33.9)	37 (21.5)	0.057	9 (64.3)	48 (22.1)	0.001*
Operative mortality	2 (3.4)	2 (1.2)	0.270*	0 (0)	4 (1.8)	>0.999*
Postop MR	9 (15.3)	24 (14.0)	0.805	4 (28.6)	29 (13.4)	0.122*
Postop MS	11 (18.6)	17 (9.9)	0.075	6 (42.9)	22 (10.1)	<0.001
MV reoperation	5 (8.5)	9 (5.2)	0.356*	3 (21.4)	11 (5.1)	0.043*

Values are presented as number (%) or median (range).

* Fisher’s exact test.

EchoCG: echocardiography; MR: mitral regurgitation; MS: mitral stenosis; MV: mitral valve; Postop: postoperative.

## DISCUSSION

To the best of our knowledge, this study is the first attempt to predict the poor MV exposure encountered in EMVS. We observed that out of 263 patients who underwent EMVS, 60 (22.8%) exhibited poor MV exposure (grade 2 + 3). Among them, approximately, a quarter were identified to have severe annular deformation caused by squeezing (grade 3). Although this study only targeted patients who underwent EMVS, the results of this study are expected to have significance for all types of minimally invasive MV surgeries, including robotic and direct-vision mini-MV surgery. Although the issue of suboptimal MV exposure during EMVS has been raised previously [4, 5, 12, 13], few studies have proposed its clear definition, incidence and impact on surgery, largely due to the difficulty in objectively defining poor exposure. However, in our study, the surgical field of view could be recorded and analysed retrospectively by multiple reviewers owing to the use of totally endoscopic surgery. Surgical video analysis is expected to have a prominent role in studies on a valve morphology because of the ability to objectively and postoperatively evaluate the operative findings, which previously depended on the subjective and on-site judgement of the operator.

In this study, 3 main criteria were used to grade the degree of MV exposure: (i) whether the annulus is obscured by the atrial wall or has deformation, (ii) whether this significantly hinders the progress of the surgery and (iii) whether there is severe squeezing of the valve that makes normal valve analysis impossible. When the surgical videos were reviewed based on this criterion, grades 0 and 3 were intuitively and easily distinguishable, while the remaining cases were classified into grades 1 and 2 as the second criterion. As the second criterion was somewhat arbitrary, if the distinction was ambiguous, it was classified as grade 1.

Our results suggest that MV exposure in EMVS affects the clinical outcome of MV repair. The rates of overall adverse outcomes, postoperative MS and MV reoperation were significantly higher in the grade 3 exposure group, while the rates of overall adverse outcome (33.9% vs 21.5%, *P* = 0.057) and postoperative MS (i.e. mean pressure gradient >5 mmHg; 18.6% vs 9.9%, *P* = 0.075) also tended to be higher in the grade 2 + 3 exposure group. These results suggest that sternotomy conversion should be considered when the MV exposure is poor, especially in the case of grade 3, while also showing that the degree of MV exposure should be considered as a factor influencing the MV repair outcome of EMVS. Additionally, these findings suggest that the effort to predict poor exposure before surgery is meaningful, especially in the case of inexperienced surgeons.

Our prediction model can be used to calculate an individual’s probability of poor MV exposure using BMI and 4 thoracic dimensions measured from preoperative CT images. However, as the frequency of grade 3 exposure was low (14 cases, 5.3%) in this study group, logistic regression analysis for its prediction had to be performed with a single variable; therefore, there were limitations in creating a predictive model. A practical prediction model with higher predictability is expected to be obtained if additional predictors such as BMI can be included in a larger study group in the future.

Which anatomical structures affect MV exposure, and in what way? This question represents another formulation of the query regarding how to address poor MV exposure. While we identified BMI and 4 CT measurements as predictors of grade 2 + 3 exposure, providing a clear answer to that question remains challenging. However, it does appear evident that the recoiled diaphragm pushes the empty heart upward during surgery, leading to atrial wall collapse and annular deformation. This inference is supported by the observation that BMI and diaphragm levels were higher in the poor exposure group. The heightened risk of poor exposure with the decreased LV apex angle can also be explained by the fact that the direction of the diaphragm’s upward pressing force becomes more parallel to the plane of the MV annulus, thereby more directly affecting the MV annular shape. Interestingly, Δdiaphragm was confirmed as a predictor in both grade 2 + 3 and grade 3 exposures. Considering that CT scans are conducted with breathing paused during end-inspiration, a higher Δdiaphragm (i.e. greater elevation of the right diaphragm compared to the midline diaphragm) seems to indicate higher intraabdominal pressure. Consequently, the diaphragm is more likely to shift upward during general anaesthesia.

### Limitations

Regarding the study limitations, some of our CT measurements may have arbitrary definitions and may not be familiar to readers. To our knowledge, there has been no previous research proposing CT measurements for predicting poor MV exposure, and all the CT measurements defined in this study were created by the authors without relying on existing references. We believe that as understanding of the thoracic anatomical structures influencing poor MV exposure deepens in the future, new CT measurements may be defined. In addition, there were missing values in CT measurements due to the differences in the CT protocols used, given the retrospective nature of this study. We did not impute these data as we are confident that no selection bias occurred, as local CT protocol was not associated with other patients’ characteristics. Consequently, only 62.4% (164 cases) of the study subjects were actually included in the stepwise regression analysis to identify predictors of grade 2 + 3 exposure, and only the 73.4% (193 cases) of the cases had Δdiaphragm input for univariate logistic analysis. As these missing values may have affected the results, additional studies with a unified test protocol are needed.

Another limitation is the short follow-up period, which was insufficient to evaluate the outcome after MV repair. In particular, out of 231 patients who underwent MV repair, 93 either had only predischarge echocardiography or did not undergo postoperative echocardiography. This situation may introduce errors in the surveillance of postoperative MR or MS. In our tertiary hospital, if patients are stable after surgery, they are referred to local hospitals for follow-up. However, if an abnormal finding is identified in postoperative echocardiography, or if patients are referred again for cardiac issues, they receive follow-up care at our hospital with reintervention if deemed necessary. Therefore, it is highly likely that almost all adverse events have been included in the analysis. However, it is necessary to fully understand that not all adverse outcomes after surgery may have been included in the analysis results for the outcome of MV repair in this study.

Additional to above, this cohort includes the learning curves of our programme and mixed pathologies, for which the clinical comparative data should be interpreted with caution. Caution is also necessary when applying these results to all EMVS, given that the method for exposing the MV varies depending on the surgeon, potentially resulting in a differing pattern of poor exposure. Therefore, additional research by other researchers on MV exposure in EMVS is needed to validate the results of this study and to determine the best approach or tool for MV exposure.

## CONCLUSION

Our study demonstrated that poor MV exposure was encountered in approximately one-fourth of the performed EMVS procedures, among which severe annular deformation caused by valve squeezing was found in one-fourth and was associated with higher adverse outcomes. Poor MV exposure might be predicted preoperatively using patients’ BMI and CT measurements, assisting in the determination of the surgical approach.

## Supplementary Material

ezae070_Supplementary_Data

## Data Availability

The data underlying this article will be shared upon reasonable request to the corresponding author.

## Notes

Presented at the 37th Annual Meeting of the European Association for Cardio-Thoracic Surgery, Vienna, Austria, 4–7 October 2023.

## ABBREVIATIONS

AUC: Area under the curve
BMI: Body mass index
CI: Confidence interval
CT: Computed tomography
EMVS: Endoscopic mitral valve surgery
LV: Left ventricle
MR: Mitral regurgitation
MS: Mitral stenosis
MV: Mitral valve
MVR: Mitral valve replacement
