# Supplementary material for: Prediction of poor exposure in endoscopic mitral valve surgery using computed tomography
Source: Eur J Cardiothorac Surg. 2024 Feb 28;65(3):ezae070. doi: 10.1093/ejcts/ezae070 (PMC10927309; doi:10.1093/ejcts/ezae070)
Supplement: ezae070_Supplementary_Data [file ezae070_supplementary_data.zip › Supplementary Tables S1 to S3 and Figure S1 & S2 legend.docx]

**Supplementary Figure Legend**

Supplementary Figure S1. Four computed tomography measurement predictors of poor mitral valve exposure

Supplementary Figure S2. Calibration plot of the final model

Supplementary Table S1. Pairs of highly correlated measurements and selected variables for logistic regression analysis

| Pairs of measurements | Correlation coefficient | Selected variable(s) for  logistic regression analysis |
| --- | --- | --- |
| Weight & BMI | 0.794 | BMI |
| Chest width-axial & chest width-coronal | 0.889 | Chest width-axial |
| Chest AP-axial & mid AP-axial | 0.902 | Chest AP-axial  or  Mid AP axial |
| Chest AP-axial & short heart-axial | 0.723 |  |
| Chest AP-axial & chest AP-sagittal | 0.966 |  |
| Chest AP-axial & mid AP-sagittal | 0.911 |  |
| Mid AP-axial & short heart-axial | 0.732 |  |
| Mid AP-axial & chest AP-sagittal | 0.898 |  |
| Mid AP-axial & mid AP-sagittal | 0.966 |  |
| Short heart-axial & chest AP-sagittal | 0.750 |  |
| Short heart-axial & mid AP-sagittal | 0.744 |  |
| Chest AP sagittal & mid AP-sagittal | 0.919 |  |
| Chest height-coronal & chest height-sagittal | 0.825 | Chest height-coronal |
| Chest height-coronal & right diaphragm | 0.785^*^ |  |
| Chest height-sagittal & right diaphragm | 0.713^*^ |  |
| Long heart-axial & heart width | 0.833 | Long heart-axial  or  Heart width |
| Mid diaphragm & right diaphragm | 0.838^*^ | Mid diaphragm |

* Spearman’s rank correlation coefficient. The others are Pearson’s correlation coefficients. AP: Anteroposterior dimension; BMI: Body mass index.

Supplementary Table S2. Combinations of variables and AUC of each variable set

| # | Variables | | | No. of cases | AUC |
| --- | --- | --- | --- | --- | --- |
| 1 | Chest AP-axial, heart width | + | BMI  Chest width-axial  Chest height-coronal  LV apex angle  Mid diaphragm  Δdiaphragm | 164 | 0.822 |
| 2 | Chest AP-axial, long heart-axial |  |  | 164 | 0.798 |
| 3 | Chest AP-axial |  |  | 164 | 0.798 |
| 4 | Mid AP-axial, heart width |  |  | 164 | 0.822 |
| 5 | Mid AP-axial, long heart-axial |  |  | 164 | 0.798 |
| 6 | Mid AP-axial |  |  | 164 | 0.798 |

“No. of cases” is the number of cases in which all values for the variable set could be obtained.

AP: Anteroposterior dimension; AUC: Area under the curve; BMI: Body mass index; LV: Left ventricle.

Supplementary Table S3. Univariate logistic regression for the prediction of grade 3 exposure

| Variables | No. of cases | OR (95% CI) | P-value | AUC | P-value |
| --- | --- | --- | --- | --- | --- |
| BMI | 263 | 1.155 (1.042–1.281) | 0.006 | 0.744 | <0.001 |
| Chest width-axial | 251 | 1.014 (0.989–1.039) | 0.286 | 0.579 | 0.388 |
| Chest AP-axial | 254 | 0.997 (0.972–1.021) | 0.782 | 0.526 | 0.656 |
| Chest height-coronal | 174 | 0.976 (0.952–1.001) | 0.059 | 0.721 | 0.001 |
| LV apex angle | 195 | 0.911 (0.832–0.998) | 0.046 | 0.711 | 0.003 |
| Heart width | 188 | 1.004 (0.962–1.047) | 0.871 | 0.474 | 0.789 |
| Mid diaphragm | 199 | 0.428 (0.182–1.005) | 0.051 | 0.709 | 0.032 |
| Δdiaphragm | 193 | 1.064 (1.015–1.115) | 0.010 | 0.826 | <0.001 |

“No. of cases” is the number of cases in which values for the variable could be obtained. AP: Anteroposterior dimension; AUC: Area under the curve; BMI: Body mass index; CI: Confidence interval; LV: Left ventricle; OR: Odds ratio.
