# Supplementary figures and images for: Prediction of poor exposure in endoscopic mitral valve surgery using computed tomography
Source: Eur J Cardiothorac Surg. 2024 Feb 28;65(3):ezae070. doi: 10.1093/ejcts/ezae070 (PMC10927309; doi:10.1093/ejcts/ezae070)

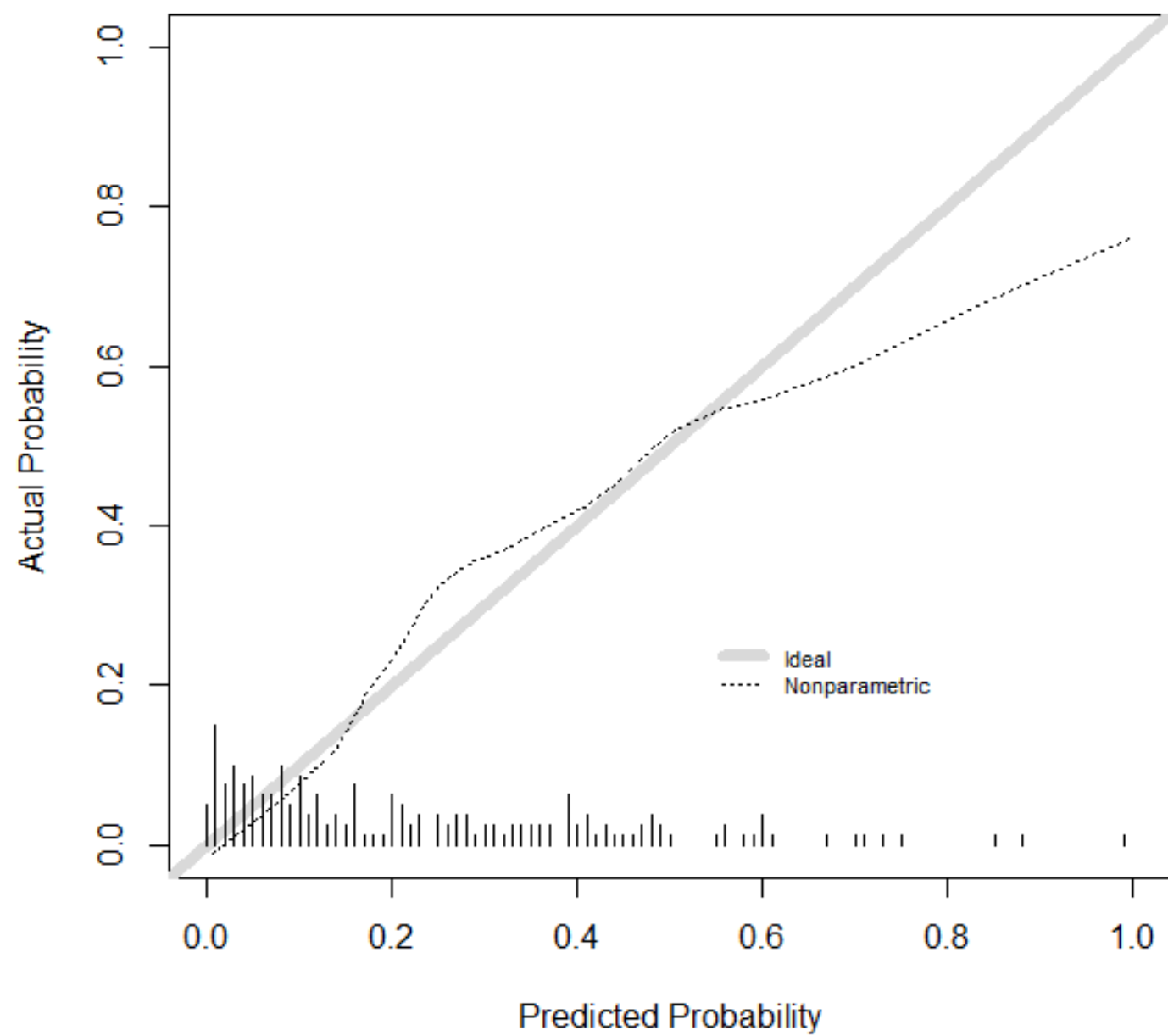

Supplement: ezae070_Supplementary_Data [file ezae070_supplementary_data.zip › Supplementary Figure S2.pdf]

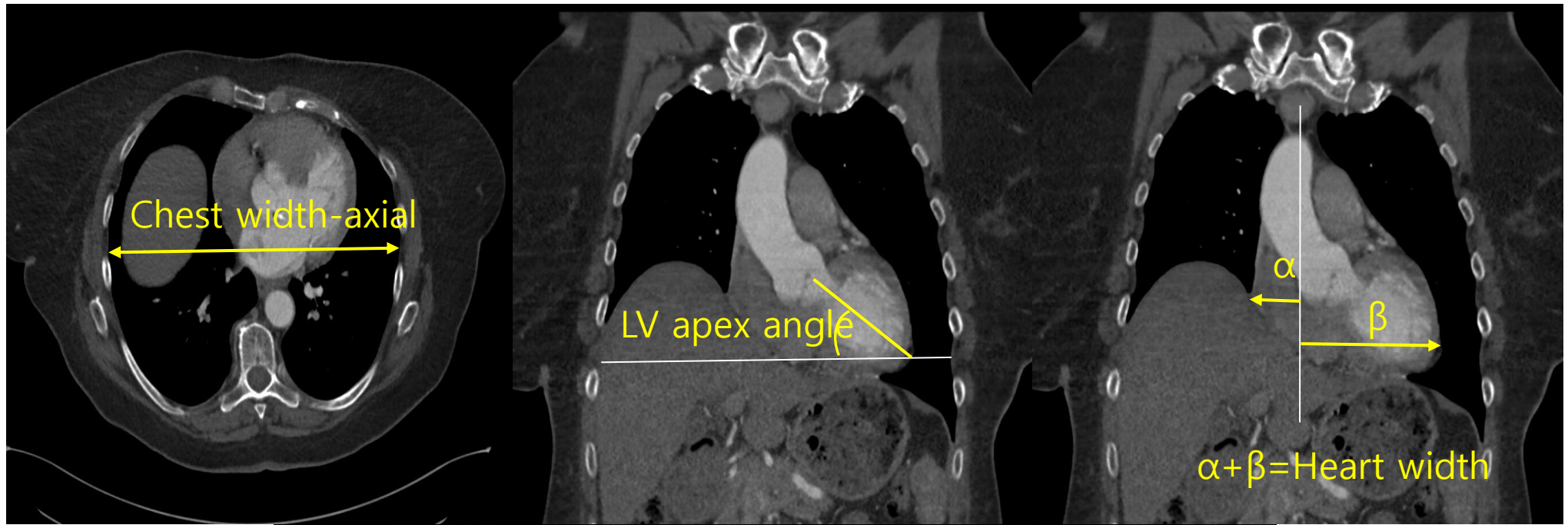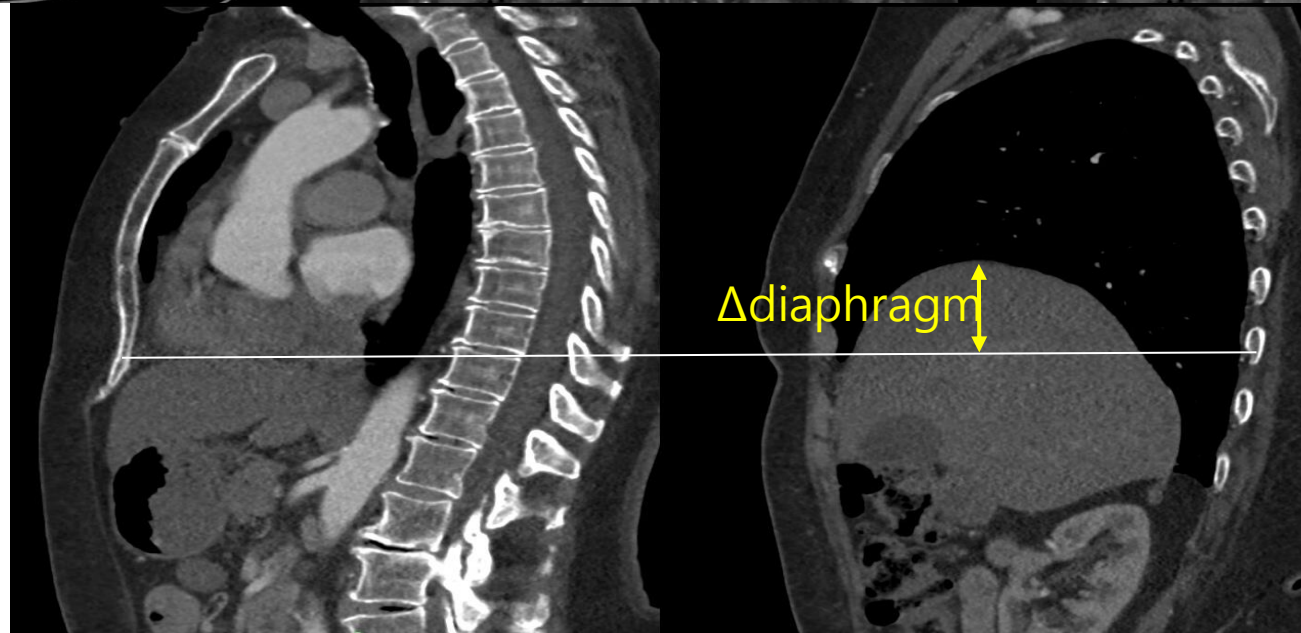

Supplement: ezae070_Supplementary_Data [file ezae070_supplementary_data.zip › Supplementary Figure S1.pdf]
